# Do 21st-century skills make you less lonely? The relation between 21st-century skills, social media usage, and students’ loneliness during the COVID-19 pandemic

**DOI:** 10.1016/j.heliyon.2024.e25899

**Published:** 2024-02-06

**Authors:** T.S. Cristea, C. Snijders, U. Matzat, A. Kleingeld

**Affiliations:** Eindhoven University of Technology, Department of Industrial Engineering and Innovation Sciences, Atlas 9.407, Groene Loper 3, 5612, AE, Eindhoven, the Netherlands

**Keywords:** COVID-19, 21st-century skills, Social media, Online contacts, Loneliness, Well-being

## Abstract

21st-century skills are a new category of competencies recommended for people to adapt to the digital era. Digital communication skills, particularly, are regarded as an important facet in a progressively moving online society. Empirical evidence of their actual value, however, is largely missing. Recently, the COVID-19 pandemic offered an appropriate testing ground for the possible beneficial role that such skills might play. Our results show that digital communication skills correlate with loneliness. However, social media usage, online contacts, and offline contacts only partially mediate the relationship between digital communication skills and the loneliness levels of students. In addition, we found substantial differences between the two waves of data collection and the types of contacts that influenced loneliness. While skills may have reduced loneliness, the precise mechanism of this effect needs to be clarified more. We offer suggestions for future research to examine the potential benefits of 21st-century skills.

## 1 Introduction

The current 21st-century society has been transforming into a knowledge society [1,2[1,2, p. 3], in which knowledge and ideas function as commodities, easily shared between individuals [3,4]. Such a society is expected to require a new set of skills, usually referred to as 21st-century skills, which are characterised as transversal (not tied to one field, but relevant across a variety of areas) and multidimensional (covering a broad scope) [1,3]. An interest in 21st-century skills also stems from the industry and business stakeholders, whose concern is that the workforce might need to catch up with the fast, dynamic changes in the workplace [5], tied mainly but not only to the digital environment. Even though there seems to be a consensus about the importance of 21st-century skills in our growingly complex society [5,6], their conceptualisation needs to be revised with many researchers supporting different definitions and components (ex. [1,4,7]). However, most researchers agree that communication and collaboration are essential dimensions of digital competencies.

As mentioned by Refs. [1,3,4,6], communication is invaluable in the workplace. However, while 21st-century communication refers to the overall communication needed in the workplace, not tied to the medium itself, digital communication is linked to the digital environment. This also leads to the main criticism of the 21st-century skills concept: its vagueness prompts problems in its conceptualisation and operationalisation. Moreover, different authors' use of different terms makes it even more difficult for researchers to agree on a universal measurement. The systematic literature review on concept usage by Ref. [8] clearly shows a large range of definitions regarding digital competence/digital literacy usage in higher education research. These can be defined by policies, research, or both, even if these are considered technical skills or social practices. Such differences directly affect the conceptualisation of 21st-century skills, some looking at global skills [5], while others consider digital skills measured in the specific context of a problem-solving paradigm [9]. Voogt and Roblin [3] found that when comparing the international frameworks of EU and OECD countries, the frameworks align regarding their goals and elements (high horizontal consistency). Still, their application was very different (low vertical consistency).

As we can see, the research on 21st-century skills has been slowed down by two main reasons: disagreement on the concept and components and the need for a clear measurement [10]. Our study will contribute to the solution by focusing on a clearly defined dimension of 21st-century skills and expressive digital communication and operationalising it with a previously used and tested scale with high reliability. We will further look at a real-life application of 21st-century skills and digital communication and their impact on the well-being of higher education students. While the effects of digital communication on well-being have been addressed and studied before, to our knowledge, such effects have never been researched within the context of 21st-century skills. Van Laar et al. [6] found evidence that ICT training and online contacts can predict various forms of digital communication skills. Our research differs in this regard since we look at their influence over social media communication and loneliness over multiple quartiles during the COVID-19 pandemic. This is important as society is becoming increasingly complex and moving online, and such skills are becoming more prominent and necessary [3]. University is also an inflexion point in life for everyone in many regards. Students start experimenting and joining various communities, further impacting their mental well-being. Students use social media platforms as an extension of school work, such as they are often called ‘third spaces’, at conjunction of institutional and non-institutional managed spaces [11]. 21st-century skills could help with better use of social media, which could, in turn, increase the number of contacts, which should improve mental well-being.

### 1.1 Literature review

The COVID-19 pandemic has forced educational institutions to move online earlier than expected [12,13], which offers a perfect background for testing the value of digital communication skills, one of the prominent and universally included aspects of 21st-century skills, among students. Most faculty staff and administrators likewise acknowledge that one of the main concerns and focuses should be the support students need to best adapt to the online environment [12]. Aristovnik et al. [13] ran the most extensive survey we know of during the first wave of the COVID-19 pandemic with higher education students. They found that students were confident in their social media usage skills but needed to be more confident about their overall digital communication skills.

One of the main usages of digital communication skills occurs on social media and online communication platforms, which allow people to create and share content and connect and build networks without physical interactions [14]. Online communication has become invaluable for staying in touch with and updating family/friends and gathering information during lockdowns [15]. Online communication and collaboration have become more ubiquitous than ever, and social media platforms (e.g., Facebook, WeChat, Twitter) are some of the world's biggest and most influential companies. Other platforms that focus entirely on online communication (such as Zoom, Whatsapp, or Microsoft Teams) have become the only way to stay in touch with colleagues and educators. These have become essential tools during the COVID-19 pandemic, in which higher educational institutions had to quickly adapt to local government's restrictions, allowing them to keep their educational activities running. While digital skills, especially through social media, have recently become increasingly important in our society, such changes have made them essential for students to participate and thrive in a modern educational institution. Greenhow and Robelia [16] looked at how technological fluency and digital citizenship relate to MySpace (one such social platform). The authors were interested in how the platform itself can act as an informal training ground for digital skills and identity formation. The results seem promising: social platforms can act as catalysts for acquiring digital skills through informal (‘learning by doing’) or non-formal (‘asking for help’) learning. Similarly [17], ran a qualitative study and identified the students' use of their digital skills when learning independently through YouTube. The students across multiple platforms often share video clips from the platform and can help create an independent community with its own social bonds outside the classroom. In this case, students used a range of skills and used both informal and non-formal learning.

The COVID-19 pandemic has also led to a significant decrease in well-being levels [18]. This has been confirmed by Ref. [19] for a sample of Bangladeshi students but has also been observed in student samples from Hong Kong, the United Kingdom, Spain, the United States, and Denmark [20]. Loneliness, one dimension of well-being, has increased due to a lack of face-to-face interactions, given the lockdowns and restrictive policies. For instance Ref. [21], found that the COVID-19 pandemic has led to higher levels of loneliness and lower well-being in a sample of Swiss older adults. For these older adults, satisfying social relations/communication acted as a buffer against loneliness during the pandemic. Gullo et al. [22] also found that higher social support, seen as a coping mechanism, has led to lower levels of depression and anxiety. For such reasons, social media has been argued to be a potentially important tool for people's mental health, especially in younger populations [23]. Active media usage, creating content and sharing with other users, can lead to higher social capital -resources that can be accessed via one's contacts- and act as emotional support that could lead to higher levels of well-being [20,22,24,25]. Similarly [26], found that adolescents used social media as a coping mechanism to deal with feelings of anxiety during the COVID-19 pandemic. Thus, there is good reason to expect that students during the pandemic use social media communication to maintain or improve their well-being and that those with more digital skills do better.

Our study examines the relationship between digital communication skills and loneliness in students in higher education during the COVID-19 pandemic. As described above, some studies have examined social media communication and well-being. Also, some studies have looked at 21st-century skills and how they are used within social media platforms [16,17]; however, their focus was on the nature of learning and acquisition of said skills. Our study will differ as it will focus on research examining whether and how digital communication skills relate to social media communication and, thereby, might be beneficial for reducing loneliness throughout the pandemic.

As previously mentioned, there have been many attempts at operationalising 21st-century skills; however, these still need to be meticulously developed [10]. More recently [5], developed a global rating scale for 21st-century skills, which lacks the necessary specificity for our study; the scale's communication dimension does not focus on the digital environment. Similarly, although measured in the digital environment, the ICT literacy measure of [9] is a similar global rating since it does not make a clear distinction between dimensions as it faces students with one large task they must solve together as a team. Thus, due to its specificity and high reliability, our study will use the dimension proposed and previously used by Ref. [6], expressive digital communication skills. We will be using data gathered at two moments: first, at the start of the COVID-19 pandemic, when lockdowns were being implemented, and second, six months later.

**Fig. 1:**
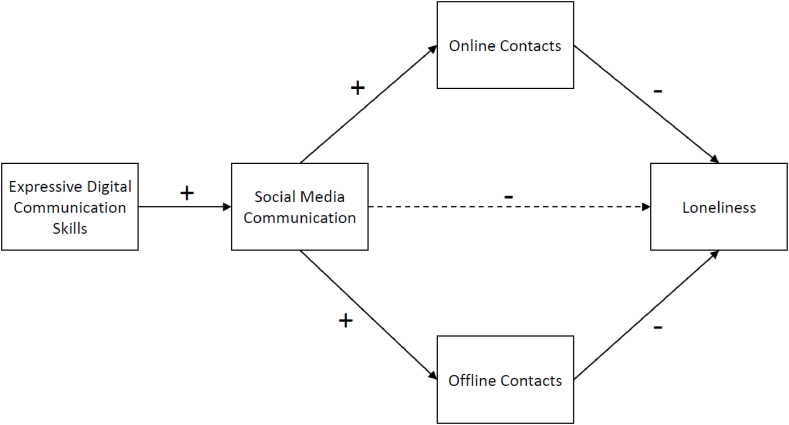
Hypothesised model representing the effect of expressive digital communication skills, social media communication, online, and offline contacts on loneliness.

Fig. 1 represents the model that we will analyse. The paper will focus on the following research question: How are expressive digital communication skills related to social media communication, online and offline contacts, and loneliness? The main application of expressive digital communication skills occurs on social media, thus our first hypothesis:

H1 Expressive digital communication skills positively relate to social media communication.

People actively using social media and engaging in more social online activities are expected to have more contacts online, which can also translate to offline contacts. Thus, our second hypothesis:

H2 Social media communication is expected to have a positive relation with online and, via a spill-over effect, also with offline contacts:

As found in Ref. [24], higher social capital can act as an emotional buffer and lead to higher well-being; thus, our 3rd hypothesis:

H3 Online and offline contacts are proposed to have a direct negative relation with loneliness, mediating the effect of social media communication.

Finally, we expect such digital communication skills to increase social media usage, which translated to higher social capital would lead to reduced loneliness:

H4 For the overall model, we expect that expressive digital communication skills have a negative relation with loneliness through social media communication, but also through online and offline contacts.

In order to answer the research question, the paper is structured as follows. In the next section, we present the methodology used. After that, we show the results and answer the research question. Finally, we discuss the findings, present limitations, and directions for future research.

## 2 Method

### 2.1 Participants and procedure

Participants were recruited using a random sampling method and included all students of one faculty (three bachelor's and four master's programs) at a Dutch university; informed consent was obtained from the participants at the beginning of the survey. We used two waves of data collection. The first online questionnaire was sent at the end of June 2020 to a total of NQ4 = 579 students, gathering data from the 4th quartile of the 2019–2020 academic year with a response rate of 37% (nQ4 = 214). The average age was M = 22,6 (SD = 2,4), with 127 identifying as male (59,3%) and 87 (40,7%) identifying as female. A total of 206 students were part of the Dutch program (96,3%), with 8 (3,7%) part of the international program. A total of 109 (50,9%) of the students had both parents with university degrees, and 43 (20,1%) had no parents with university degrees. In comparison, 56 (26,2%) had only their mothers, and 6 (2,8%) had only their fathers with university degrees.

The second wave of data collection took place in October 2021 and was sent to NQ1 = 1778, gathering information about the 1st quartile of the 2020–2021 academic year, again with a response rate of 37% (nQ1 = 658). The average age was M = 21,9 (SD = 2,3), with 365 identifying as male (55,5%), 292 (44,4%) identifying as female, and 1 (0,1%) identifying as other. A total of 610 students were part of the Dutch program (92,7%), and 48 (7,3%) were part of the international program. A total of 335 (50,9%) of the students had both parents with university degrees, 135 (20,5%) had neither, while 141 (21,4%) had only their mothers and 47 (7,2%) had only their fathers having a university degree. Over both quartiles, we had 872 completed surveys; for administrative reasons, we had to select a smaller sample of students for Q4 2019–2020. The overlapping sample was nQ4Q1 = 85.

Most courses were online for the 4th quartile of the 2020–2021 academic year. During Q1 of the 2020–2021 academic year, the measures were starting to loosen up. This allowed the faculty to schedule some of the courses on campus. Although the aim was to provide at least one on-campus activity for each student group, practical restrictions, such as the 1.5-m distancing rule, caused most educational activities to be still carried out online.

The Ethical Review Board of the Human-Technology Interaction Group at Eindhoven University of Technology approved the study; approval number 1133. Each participant was asked for consent for collecting the data and participating in the study. All data was anonymized for the analyses. A grant from the Dutch 4TU has funded the data collection.CEE (4 TU Center for Engineering Education) to Matzat, Kleingeld and Snijders (grant number IF2020-Matzat- Kleingeld-Snijders).

### 2.2 Measures

#### 2.2.1 Expressive digital communication skill

Expressive digital communication skill was measured using three items: ‘At your study (or work): how often’ … ’, ‘ … do you get what you want from interactions on the internet?’, ‘ … are you effective in accomplishing what you want from others, via the internet?’, ‘ … do you know how to use the internet to express ideas clearly?’. The items were measured on a 1–5 Likert scale, with a Cronbach's alpha of .75 for the whole sample. These items were taken from Ref. [6], with an original alpha of .79. Expressive digital communication skills can be defined as the ability of a person to derive satisfactory outcomes from online interactions [6, p. 9], which may thereby facilitate social media communication. We chose this component of the 21st-century skills since, as mentioned earlier, digital communication is seen as a vital skill to have. At the same time, their expressiveness allows us to operationalise it better and measure it.

#### 2.2.2 Social media communication

Social media communication was measured using three items, developed by the authors: ‘After the transition, I used social media (Whatsapp, Facebook etc.) intensively to stay in touch with my fellow students’, ‘ … with my friends outside university’, ‘ … with my family’. The items were measured on a 1–7 Likert scale, with a Cronbach's alpha of .85 for the whole sample.

#### 2.2.3 Online and offline contacts

Online and offline contacts were measured using three items for each construct on a 1–7 Likert scale; the variables were formed from the means of their three items. For online contacts, the items were: ‘How often did you have offline contact with the following (types of) persons during Q4 (or Q1)?’: ‘Fellow students’, ‘Family’, ‘Friends’. The same items were used for offline contacts, replacing the term online with offline. The items did not measure facets of a construct but rather interactions with different groups of people that are not necessarily positively associated with each other (e.g., a student may have many online contacts with fellow students but few with family because the student is still living at home). Social media communication and the number of online/offline contacts measured students' relation to the same people: colleagues from university, family, and friends, allowing us to use both (simultaneously) in our model. The mean values of these items can be found in Table 1 in the Results section.

**Table 1:** Descriptive Results for both Quartiles.

*Variable*	*Q4 2019–2020 (n* = *214)*		*Q1 2020–2021 (n* = *658)*	
	M	SD	M	SD
*Skills*	3.72	0.68	3.63	0.66
*Social Media Communication*	5.17	1.34	5.13	1.29
*Online contacts*	5.04	1.19	4.63	1.40
*Offline contacts*	3.80	1.11	4.15	1.06
*Loneliness*	2.06	0.65	2.12	0.64

#### 2.2.4 Loneliness

Loneliness was measured using eight items from the Revised UCLA scale questionnaire [27]. The items are:’ I lacked companionship.’, ‘I did not feel alone.’ (R), ‘I felt in tune with the people around me.’ (R), ‘I felt left out.’, ‘I felt isolated from others.’, ‘I could find companionship when I wanted it.’ (R), ‘I was no longer close to anyone.’, ‘I felt part of a group of friends.’ (R). The items were measured on a 1–4 Likert scale, with a Cronbach's alpha of .82, for the whole sample. We chose this specific scale due to its high reliability and easy interpretability.

This survey was part of a larger study on student well-being during the COVID-19 pandemic. The survey also measured other variables such as class boredom levels, goal orientation trichotomy, study crafting, and concentration. We included only the most relevant constructs related to our research question.

#### 2.2.5 Statistical analysis

All the statistical and path analyses were done using STATA 16 [28]. Descriptive analyses were used to obtain the characteristics of the sample and identify differences between quartiles. Bivariate correlations were used to assess relations between constructs. We used path analysis to test the full model.

Next, we first present the descriptive and correlational data, followed by the path analysis results. We start with the model proposed in Fig. 1. We then modify the model by removing nonsignificant relations and adding any links suggested by the modification indices and matrices of residual that are also supported by theory and make causal sense to check the robustness of the results. We follow up on the initially proposed model with models that use each of the online and offline contacts items independently to get a more detailed view of the role of each group of social contacts. Finally, we present a model without the online and offline contacts variables to analyse whether social media communication mediates the relation between expressive digital communication skills and loneliness. All results will be reported per quartile.

## 3 Results

### 3.1 Background information

Table 1 shows the expressive digital communication skills, social media communication, online/offline contacts, and loneliness means and standard deviations for each quartile. While social media usage was constant between Q4 and Q1 with very small changes in averages, the number of offline contacts significantly increased (t(870) = 4.54, p < .001), and the number of online contacts significantly decreased (t(870) = - 3.57, p < .001). This could be due to the social distancing restrictions being slowly removed between Q4 and Q1. Loneliness levels slightly increased between Q4 and Q1. Finally, expressive digital communication skills slightly decreased between Q4 and Q1, but the difference was small, similar to loneliness.

### 3.2 Correlation analyses

Table 2 shows the correlations between the variables for Q4. The most important finding is that, in line with findings in the literature, expressive digital communication skills are negatively associated with loneliness. The effect size is small to moderate. All our measurements that refer to online media positively correlate with each other (expressive digital communication skills, social media communication, and online contacts); these results support our 1st hypothesis for both quartiles and our 2nd hypothesis for Q4. Since the relation between expressive digital communication skills and offline contacts was insignificant, we cannot confirm the 2nd hypothesis for Q1. Offline contacts correlated only with online contacts, while loneliness correlated negatively with online contacts. So far, these relations were expected. However, it is important to mention that offline contacts did not significantly correlate with loneliness. This could be attributed to the lack of face-to-face meetings during this quartile. Even more so, social media communication did not correlate substantially with loneliness, the relation being non-existent.

**Table 2:** Intercorrelations between the study variables for Q4 (n = 214).

*Variables*	*1*	*2*	*3*	*4*	*5*
*1. Skills*					
*2. Social Media Communication*	.21**				
*3. Online contacts*	.29***	.46***			
*4. Offline contacts*	.07	−.02	.31***		
*5. Loneliness*	−.29***	−.01	−.19**	−.13	

*p < .05, **p < .01, ***p < .001.

Table 3 shows correlations between the variables in the subsequent quartile Q1. We find results similar to the previous quartile. Again, most importantly, students with stronger expressive digital communication skills tend to feel less lonely, with an effect size between small and moderate. Once again, the strongest relationship was found between social media communication and online contacts. Moreover, having more online or offline contacts or using more social media was associated with lower levels of loneliness. An important change is that in Q1, the variable offline contacts was significantly associated with the other variables, consistent with our hypothesis in Fig. 1.

**Table 3:** Pairwise correlations between the variables for Q1 (n = 658).

*Variables*	*1*	*2*	*3*	*4*	*5*
*1. Skills*					
*2. Social Media Communication*	.16***				
*3. Online contacts*	.21***	.35***			
*4. Offline contacts*	.19***	.09*	.28***		
*5. Loneliness*	−.34***	−.13***	−.18***	−.33***	

*p < .05, **p < .01, ***p < .001.

### 3.3 Path analysis models for Q4 and Q1

Having established the expected negative relationship between students’ expressive digital communication skills and their feelings of loneliness, we now examine the hypothesised underlying mechanisms that may explain this phenomenon.

**Fig. 2:**
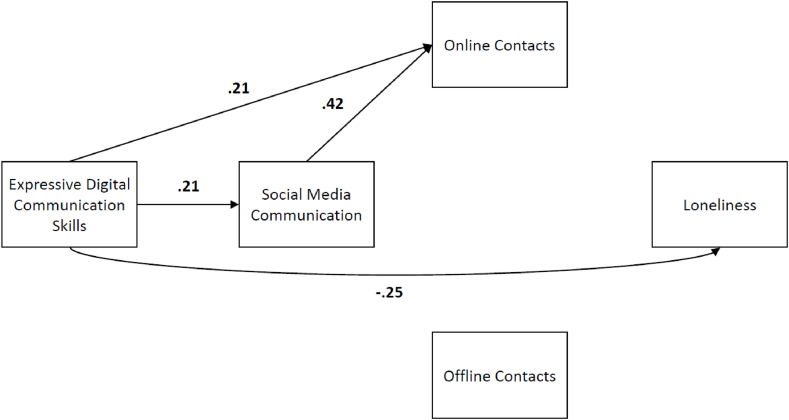
Final Model with the Significant Standardized Path Coefficients for Q4 (n = 214) *Note.* This model has been modified based on the original hypothesised model. We removed the nonsignificant relations. All shown coefficients are significantly different from zero with *p* < .05.

*Estimating the proposed model for Q4.* We found significant paths from expressive digital communication skills to social media communication, from social media communication and expressive digital communication skills to online contacts, and from expressive digital communication skills and online contacts to loneliness; both these latter relations were negative. The strongest effect was between social media communication and online contacts (β = 0.42, SE = 0.06, t = 7.61, p < .001). We can also see that the variable offline contacts did not have any significant relation with other variables in the model. However, looking at the indicators, we found a poor fit: (X^2^(1) = 27.667, p < .001), RMSEA = 0.354, pclose< .001, CFI = 0.773, and SRMR = 0.078). The matrices of residuals and the modification indices suggest that adding a covariance between the number of online and offline contacts is a viable option to improve the fit. However, offline contacts still had no significant relations within the model, so this was not possible. For the final model presented in Fig. 2, we removed all the nonsignificant relations for ease of interpretation.

*Final model for Q4*. The final model for Q4, with standardised coefficients, can be seen in Fig. 2 below. All indices suggested a close fit (X^2^(1) = 3.39, p = .066), RMSEA = 0.106, pclose = .138, CFI = 0.974, TLI = 0.843, and SRMR = 0.033). These results only partially confirmed our hypothesis of the relationship between expressive digital communication skills and loneliness. Most importantly, the role of social media communication and online contacts differed from our expectations. While expressive digital communication skills directly and significantly predicted loneliness, social media communication and online contacts did not.

*Estimating the proposed model for Q1.* We ran the same original model for the Q1 data. Compared to the previous quartile, most paths now were significantly different from zero, which may be explained by the substantially larger sample size. Looking at the indicators, we found a poor initial fit (X^2^(1) = 41.50, p < .001), RMSEA = 0.248, pclose< .001, CFI = 0.875, TLI = −0.247, and SRMR = 0.060). As in Q4, adding a covariance between the number of online and offline contacts was suggested when looking at the matrices of residuals and the modification indices. Following the same procedure as in Q4, we removed the nonsignificant relations and added the covariance.

**Fig. 3:**
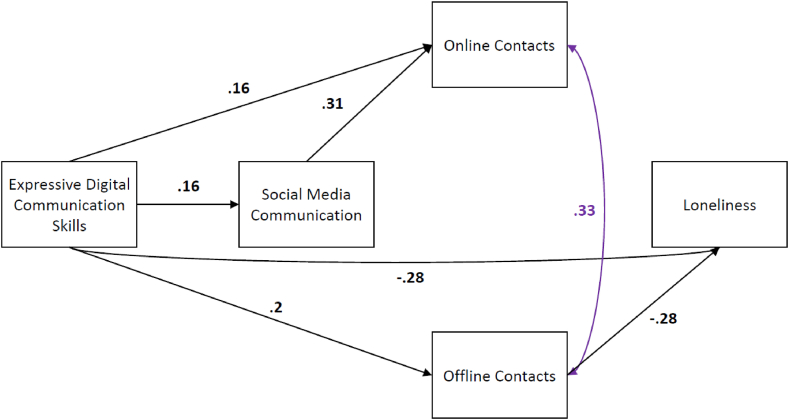
Final Model with Significant Standardized Path Coefficients for Q1 (n = 658) *Note.* This model has been modified based on the original hypothesised model. We removed the nonsignificant relations and added the covariance between online and offline contacts (purple colour-coded). All shown coefficients are significant with *p* < .05.

*Final model for Q1*. The final model for Q1, with standardised coefficients, can be seen in Fig. 3 below. All indices suggested a fitting model ((X^2^(3) = 6.32, p = .097), RMSEA = 0.041, pclose = .556, CFI = 0.990, TLI = 0.966, and SRMR = 0.029). Most importantly, and in line with the findings in the previous quartile Q4, the role of social media communication and online contacts is different than expected. We hypothesised that social media communication and online contacts would mediate the relationship between expressive digital communication skills and loneliness. Still, the relation between skills and loneliness is, to a large extent, independent of these. To a small extent, the effect is mediated by offline contacts. This mediation, however, does not show its effect via social media communication, which is outside our hypotheses.

Thus far, the findings in both quartiles are not completely in line with our expectations about how skills could predict loneliness, only partially confirming our 3rd and rejecting our 4th hypothesis. In both quartiles, online contacts did not reduce loneliness. Also, social media communication was separate from the number of offline contacts. It might be that students’ feelings of loneliness are predicted exclusively by contact with a specific type of group. In line with this idea, we repeat the analyses for the two quartiles but now distinguish the three types of contacts for both online and offline contacts. In the following post hoc analyses, we look closely at these online and offline contacts, which cover contacts with three social groups: family, friends, and fellow students. The following models replace the online and offline contact variables with the three types of contacts forming them: family, friends, and other students. Thus, every kind of contact is considered individually within the models.

**Fig. 4:**
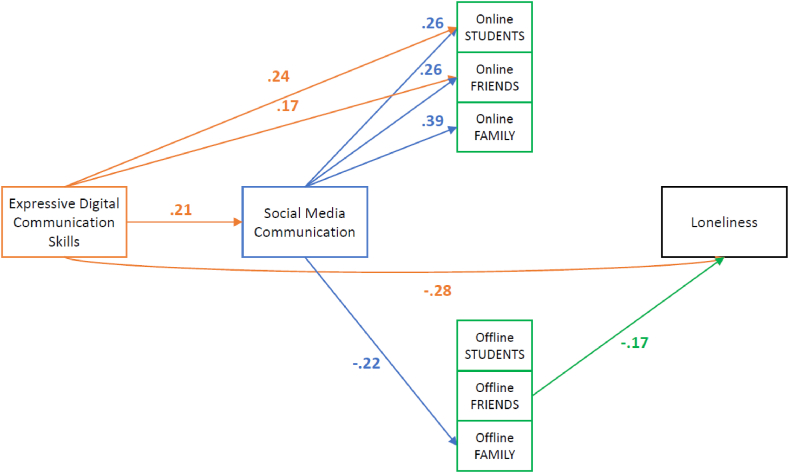
Independent Items Model for Online and Offline Contacts of Loneliness Factor Causes with Standardized Path Coefficients for Q4 (n = 214) *Note.* Colours were used to better discern between the variables and their significant paths. All shown coefficients are significant (*p* < .05). (For interpretation of the references to colour in this figure legend, the reader is referred to the Web version of this article.)

*Independent Items Model for Q4.* The final model for Q4 with the significant paths and standardised coefficients can be seen in Fig. 4 below. The model is a poor fit: ((X2(15) = 166.242, p < .001), RMSEA = 0.218, pclose< .001, CFI = 0.475, TLI = −0.261, and SRMR = 0.124).

The most important finding is that while social media communication is positively related to all three types of online contacts, none directly correlate with feelings of loneliness. This time, we can also see that social media communication does have a significant relationship with one type of offline contacts, namely offline family contacts. However, contrary to our expectations, this negative effect suggests that less social media usage leads to more offline family contact. Regarding offline contacts, we can also see that the number of offline contacts with friends negatively relates to loneliness. Regardless, we conclude that in Q4, the relationship between skills and loneliness cannot be explained by a path going via social media communication to specific types of contacts, whether online or offline.

**Fig. 5:**
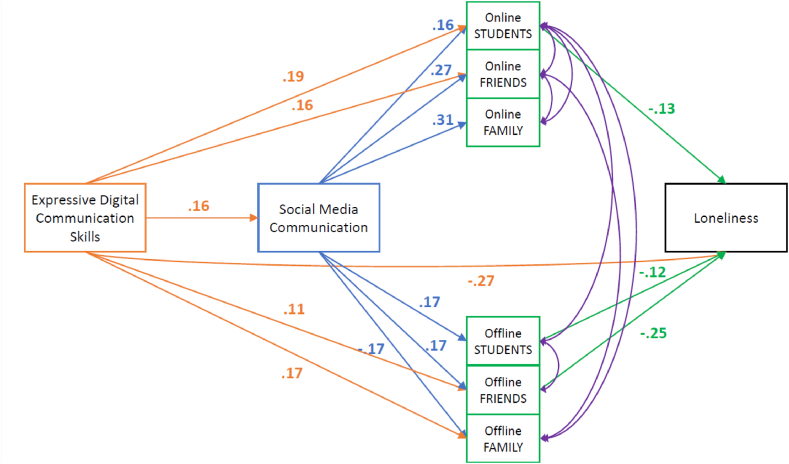
Independent Items Model for Online and Offline Contacts of Loneliness Factor Causes with Standardized Path Coefficients for Q1 (n = 658) *Note.* Colours were used to better discern between the variables and their significant paths. All shown coefficients are significant (*p* < .05). (For interpretation of the references to colour in this figure legend, the reader is referred to the Web version of this article.)

*Independent Items Model for Q1.* The final modified model for Q1 can be seen in Fig. 5 below and has a good fit: ((X^2^(15) = 507.448, p < .001), RMSEA = 0.224, pclose< .001, CFI = 0.489, TLI = −0.228, and SRMR = 0.121). The findings in Fig. 5 suggest why some expected effects did not show up in the earlier model presented in Fig. 3. For instance, Fig. 5 shows that social media communication positively relates to offline contact with fellow students but negatively to offline contact with family members. These two effects have cancelled each other out, leading to an overall nonsignificant impact of offline contacts on loneliness, as shown in Fig. 3, which masks two opposing paths.

Most importantly, the pattern of significant effects demonstrates three paths that might explain how digital communication skills may affect loneliness. First, there is a path from skills via social media communication and online contacts to fellow students to loneliness. Second, there is a path to loneliness via social media communication and offline contact with students. Third, there is a path from skills via social media communication and offline contacts with friends to loneliness. All three paths are in line with the hypothesised reasoning. However, all three paths show very small effect sizes (−.003, −0.003, and −0.007), leading to an overall effect size of −0.013, which is very small. The direct effect of skills on loneliness persists and is much larger and hence not mediated by these indirect effects of skills. It is important to mention that for this model to be a good fit, we added seven covariances based on the model's modification indices and matrices of residuals. These added relations keep the existing relations of our main variables the same.

**Fig. 6:**
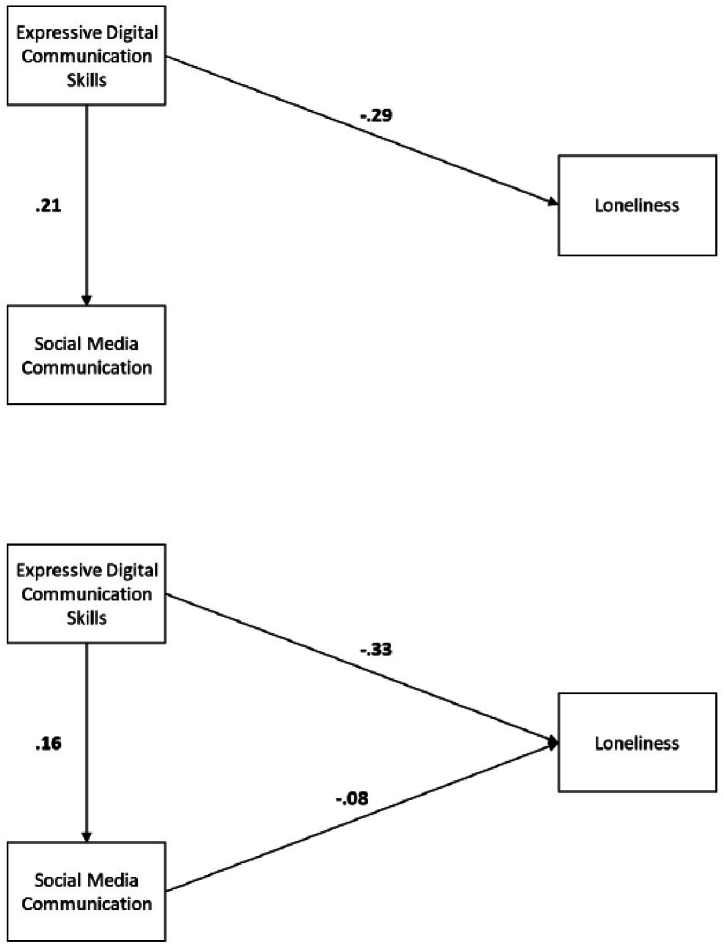
Final Modified Models Without Contacts of Loneliness Factor Causes with Standardized Path Coefficients for Q4 (above) and Q1 (below) *Note.* All shown coefficients are significant (*p* < .05).

Summarising the results of the four path models (Figs. 2–5), our hypothesised mechanism does not explain the relationship between skills and loneliness in Q4 and only to a very small extent in Q1. To see whether social media communication would play any role in the relationship between skills and loneliness, we ran a final path analysis model with social media communication as a mediator between expressive digital communication skills and loneliness, neglecting online and offline contacts. Both final models for Q4 and Q1, with standardised coefficients, can be found in Fig. 6 below. The data of Q4 confirms our expectations: social media communication has no significant relation with loneliness in the fourth quartile. The Q1 model shows a significant relation between social media communication and loneliness but with a small effect size (β = −0.08, SE = 0.04, t = −2.22, p = .026). The direct effect of skills on loneliness in Q4 is −0.33, which is almost identical to the original effect of −0.34, as shown in Table 3.

The findings in Fig. 6 align with the conclusions we draw from the earlier path analyses (Figs. 2–5). In the fourth quartile, social media communication does not explain the relationship between skills and loneliness. In the subsequent quartile 1, three specific paths can explain the loneliness-reducing effect of skills. However, these three paths have small effect sizes, whereas the original direct effect of skills persists and is much larger. Table 4 below summarises the proposed hypotheses and their verdict per dataset.

**Table 4:** Proposed hypotheses and their verdict per quartile.

Number	Hypothesis	Verdict for Q4 data	Verdict for Q1 data
H1	Expressive digital communication skills positively relate to social media communication.	Supported	Supported
H2	Social media communication is expected to have a positive relation with online and, via a spill-over effect, also with offline contacts.	Partially supported	Supported
H3	Online and offline contact are proposed to have a direct negative relation with loneliness, mediating the effect of social media communication.	Partially supported	Partially supported
H4	For the overall model, we expect that expressive digital communication skills have a negative relation with loneliness through social media communication but also online and offline contacts.	Rejected	Partially supported

## 4 Discussion

The debate about 21st-century skills, previously defined as contemporary, digital, and transversal competencies [1,3], emphasises the usefulness of these skills. Still, empirical evidence favouring beneficial effects and knowledge about underlying mechanisms that can explain *how* they work is scarce. This lack of evidence provides the rationale for our study. Our main research goal was to investigate whether and, if yes, *how* 21st-century skills affect students’ loneliness during the COVID-19 pandemic. Our study focused specifically on expressive digital communication skills, one facet of 21st-century skills defined by Ref. [1] that seems promising for facilitating online communication during a lockdown. While the effect of digital communication on well-being has been studied before [ex. 24], as far as we know, the role of specific 21st-century skills has not been studied so far. Previous research shows that contact with other people can reduce loneliness [21]. We specifically looked at three groups of people with whom higher education students have the most contact: fellow students, friends, and family. Since expressive digital communication skills focus mainly on the digital environment, we expected the period of the COVID-19 pandemic to be an appropriate moment to study this effect due to social distancing policies, which have forced most social contacts to move online.

Our results show that students with stronger expressive digital communication skills felt less lonely in both quartiles during the pandemic. In this sense, they have profited from their digital communication abilities. In addition, we aimed to reveal how skills contribute to reducing loneliness. We expected stronger skills to increase social media communication, leading to more online and offline contacts (i.e., social capital), which diminished feelings of loneliness. Our first two presented hypotheses studied parts of the overall mechanism. Overall, we did find a positive relation between expressive digital communication skills and social media communication (supporting H1) and between social media communication and contacts, partially supporting H2 for Q4 and fully for Q1. Also, the proposed mediation model of contacts between social media usage and loneliness was partially supported (H3). However, for the first period that we studied the data (quartile 4 of 2020–2021), we did not find evidence that supported the entire hypothesised mechanism in any way (rejecting H4 for Q4). For the second period that we studied (quartile 1 of 2021–2022, the next academic year), we found three different paths that related skills via increased social media communication to reduced loneliness (partially supported for Q1). Social media communication increased online communication with fellow students, offline communication with fellow students, and offline communication with friends, all of which decreased feelings of loneliness slightly. However, the overall effect size of all three paths is small compared to the direct effect of skills on loneliness and does not explain it away. We conclude that our hypothesised model does not, or only to a small extent, capture the mechanisms that relate skills to less loneliness.

Some of our results, for instance, specific paths relating social media communication to reduced loneliness, align with previous findings indicating that online communication can act as social support and lead to higher well-being [24], also during the COVID-19 pandemic [20]. In the study by Ref. [29], researchers found an effect of online communication (video calling) on reducing loneliness. In this case, the residents of an elderly care home, some suffering from dementia, started having regular Skype calls with secondary school students. The study looked specifically at intergenerational friendship, which increased well-being. Researchers from Chile [30] used the national database to study the effect of school digitalisation on personal student well-being in almost 200 schools. The school's digital development was represented by ICT infrastructure, implementation of technology in education, and digital skill development programs for the teacher and students, all showing a positive relation with students' subjective and social well-being at school. This aligns with our findings, as both studies found a direct link between 21st-century skills and well-being (in our case, loneliness). The authors of the [31] study looked at important life transitions, thus considering the same study context, university years, and similar variables, social capital and loneliness. Similarly, they found a clear negative effect between higher social capital and lower levels of loneliness. When further considering the COVID-19 pandemic [32], found higher loneliness levels in younger adults during the pandemic, with higher levels of social support (i.e. social capital) being the strongest protective factor against it.

This study covered two periods to look for possible differences between online and offline contacts in students’ well-being between quartiles as face-to-face policies changed. During the fourth quartile, all university courses took place online. However, during the subsequent first quartile of the next academic year, one-third of the courses took place on campus as the restrictions were lifted. Thus, students in the fourth quartile relied almost entirely on the online environment to keep in touch and interact with peers, friends, and family, while in the subsequent quartile Q1, they were allowed to have more face-to-face meetings. The latter may explain the increase in the mean number of offline contacts between quartiles and why the correlation between offline contacts and loneliness is stronger in Q1. The different restrictions may also explain why two paths that mediate the effects of skills on loneliness via offline contacts with fellow students and friends showed up only in the later quartile but not in the earlier one. However, by and large, the divergence in findings between the two periods does not affect the general conclusion that the hypothesised model does not explain the relationship between skills and loneliness well.

### 4.1 Limitations

The study possibly suffers from sampling, response, and nonresponse bias since the survey was not mandatory, and the participants received a 5-euro incentive. Students with low 21st-century skills could have avoided such a survey due to their lack of confidence. People with low well-being (loneliness being the dimension we tested) tend to isolate themselves, which might have also affected sampling issues. Measurements of 21st-century skills are highly debated [6]. Although our choice of specific skills looked promising for student communication, other 21st-century skills (e.g., content-sharing communication) may be more valuable for reducing loneliness. The cross-sectional aspect of our study is also worth noting. A larger sample would provide more insight into such a mechanism if followed over time in a longitudinal study. However, a limitation is the nature of loneliness measures: a quartile (4 months) is a large break between such measurement points, bringing up possible validity questions.

### 4.2 Future research

Future research needs to look more closely at the environment new social media platforms create and how 21st-century skills and types of contact interact. This should also be studied within the context of well-being since the online environment has been shown to impact well-being positively, and 21st-century skills should not only be understood as instrumental for reaching learning outcomes alone. We suggest how future research could analyse the relationship between skills and loneliness in more detail.

First, we propose an explanation based on observations and the current evolution of new social media and online platforms. Our social media communication and online and offline contacts are focused solely on fellow students, friends, and family, which are known to contribute to so-called *bonding* social capital [31,33]. However, new social media platforms have started creating large communities between individuals initially meeting as strangers. Such communities contribute to bridging a person's social capital, extending far beyond an individual's family and friends and working across different networks. People do not necessarily identify such individuals as friends but feel part of a community with common interests and hobbies. The internet is unique in this regard, compared to traditional offline and face-to-face interactions, as it can widely enhance and encourage individuals to access and increase their bridging social capital. Online bridging across networks is associated with lower loneliness levels, although cultural differences were found [33]. Simply put, interactions on the internet do not need to refer to a well-known person to become effective and influence lifestyle and behaviour. A simple example is streaming platforms (such as Twitch or Discord). Those are usually used by people who have never met each other but can spend time together and form communities revolving around common interests. Such platforms promote active media usage, which has been found to increase well-being and decrease loneliness by increasing emotional support [20,24,31]. An extreme example concerns streams that show people sleeping or eating with almost no interaction. People join those to feel less lonely and be part of a community. Expressive digital communication skills may be of particular value for social communication via these broader and more fluid networks.

Secondly, another important factor could be the nature of learning. Learning can be formal, informal, or non-formal [16]. For example, regarding 21st-century skills, a person can take a course (formal), learn by doing (informal), or by asking someone (non-formally). Being part of one of the previously mentioned communities, students would have access to more experienced users; thus, non-formal learning would be more common. Such a relation would explain our results since higher non-formal education would require a stronger community (higher bridging capital), leading to lower loneliness. Further research on this is needed.

Another possible explanation refers to the complexity of the concepts. There is much debate about the exact definition of social media communication [24,34]. Social media usage can be both active and passive, but also one way or two ways [34]. When contacting a fellow student, friend, or family, an individual engages in more active two-way communication. However, when building bridging social capital, the platform type becomes essential as different platforms promote and use different types of interaction between users and creators. Users can be both active and passive. This further interacts with the community size and the goals of the community. Further research needs to be done to understand how the community, online skills, and type of social media usage interact and affect well-being.

There is an effect of 21st-century skills, expressive digital communication to be precise, on loneliness. While we did not identify the exact mechanism behind the relation, such a relation does exist, and we strongly believe it should be further investigated. We offer multiple explanations for the direct effect of 21st-skills on loneliness that range from the influence of the current social media landscape to the complexity of the concept and its multitude of sub-dimensions.

## 5 Conclusion

Our two quartile analyses show that students with stronger 21st-century skills felt less lonely during the COVID-19 pandemic. We thereby contribute to the limited evidence on the beneficial effects of 21st-century skills. Moreover, skills' impact on loneliness was only to a very limited extent mediated by increased social media communication and growth of communication with fellow students online or offline or via more offline communication with friends. While our fully proposed model still needs to be confirmed, the positive effect that digital skills can have on well-being is important and should be further investigated. We offer multiple explanations for this, including the presence of strictly online communities that students can be part of. Such communities are an effect of modern online presence, and they can greatly impact well-being and loneliness, although their exact mechanism is not completely understood yet. Further explanations refer to the nature of such digital skills, their problematic measurement in the literature, and how students can use social media. Future research should look at how the application of these skills affects social media platforms' use and well-being in general. Such knowledge is valuable as it demonstrates the relevance of specific, well-defined, measurable forms of 21st-century- skills.

## Data availability statement

Sharing research data helps other researchers evaluate your findings, build on your work and to increase trust in your article. We encourage all our authors to make as much of their data publicly available as reasonably possible. Please note that your response to the following questions regarding the public data availability and the reasons for potentially not making data available will be available alongside your article upon publication.

Has data associated with your study been deposited into a publicly available repository?

Answer: No.

Sharing research data helps other researchers evaluate your findings, build on your work and to increase trust in your article. We encourage all our authors to make as much of their data publicly available as reasonably possible. Please note that your response to the following questions regarding the public data availability and the reasons for potentially not making data available will be available alongside your article upon publication.

Has data associated with your study been deposited into a publicly available repository?

Answer: The authors do not have permission to share data.

## CRediT authorship contribution statement

**T.S. Cristea:** Writing – review & editing, Writing – original draft, Visualization, Software, Formal analysis, Data curation. **C. Snijders:** Writing – review & editing, Validation, Supervision, Resources, Project administration, Funding acquisition, Conceptualization. **U. Matzat:** Writing – review & editing, Supervision, Resources, Project administration, Methodology, Investigation, Funding acquisition, Conceptualization. **A. Kleingeld:** Writing – review & editing, Validation, Supervision, Resources, Project administration, Methodology, Investigation, Funding acquisition, Conceptualization.

## Declaration of competing interest

The authors declare that they have no known competing financial interests or personal relationships that could have appeared to influence the work reported in this paper.
